# Transcriptome profiling with focus on potential key genes for wing development and evolution in *Megaloprepus caerulatus*, the damselfly species with the world's largest wings

**DOI:** 10.1371/journal.pone.0189898

**Published:** 2018-01-12

**Authors:** Wiebke Feindt, Sara J. Oppenheim, Robert DeSalle, Paul Z. Goldstein, Heike Hadrys

**Affiliations:** 1 University of Veterinary Medicine Hannover, ITZ—Division of Ecology and Evolution, Hannover, Germany; 2 Leibniz University Hannover, Hannover, Germany; 3 American Museum of Natural History, Sackler Institute for Comparative Genomics, New York, NY, United States of America; 4 Systematic Entomology Laboratory (USDA-ARS), National Museum of Natural History, Washington, DC, United States of America; 5 Yale University, Department of Ecology & Evolutionary Biology, New Haven, Connecticut, United States of America; Universitetet i Bergen, NORWAY

## Abstract

The evolution, development and coloration of insect wings remains a puzzling subject in evolutionary research. In basal flying insects such as Odonata, genomic research regarding bauplan evolution is still rare. Here we focus on the world’s largest odonate species—the “forest giant” *Megaloprepus caerulatus*, to explore its potential for looking deeper into the development and evolution of wings. A recently discovered cryptic species complex in this genus previously considered monotypic is characterized by morphological differences in wing shape and color patterns. As a first step toward understanding wing pattern divergence and pathways involved in adaptation and speciation at the genomic level, we present a transcriptome profiling of *M*. *caerulatus* using RNA-Seq and compare these data with two other odonate species. The *de novo* transcriptome assembly consists of 61,560 high quality transcripts and is approximately 93% complete. For almost 75% of the identified transcripts a possible function could be assigned: 48,104 transcripts had a hit to an InterPro protein family or domain, and 28,653 were mapped to a Gene Ontology term. In particular, we focused on genes related to wing development and coloration. The comparison with two other species revealed larva-specific genes and a conserved ‘core’ set of over 8,000 genes forming orthologous clusters with *Ischnura elegans* and *Ladona fulva*. This transcriptome may provide a first point of reference for future research in odonates addressing questions surrounding the evolution of wing development, wing coloration and their role in speciation.

## Introduction

The bauplan evolution of the Pterygota (flying insects) is one of the major challenging subjects of evolutionary research. Although the unique appearance of wings in Hexapods has led to the greatest adaptive radiations in the animal kingdom, the precise developmental mechanisms and their evolution are yet not fully understood [1]. A wide range of research is focusing on wing development, shape and coloration and their role in speciation, but so far most research has been limited to more derived model systems such as *Drosophila* sp., *Tribolium* sp. and some Lepidoptera [1–7].

Today progress in high throughout sequencing, advancing analytical methods and an easy access to next generation sequence data, makes integrative approaches achievable for non-model organisms [7–10]. Specifically, transcriptomics are suitable because they enable simultaneously the analysis of expression patterns of known developmental genes and the identification of new candidate genes [8, 11]. Moreover, interspecific transcriptome comparisons enhance our ability to infer the mechanisms underlying homologous structural and functional changes as well as allow to detect fundamental principles and conserved features [12, 13].

**Fig 1:**
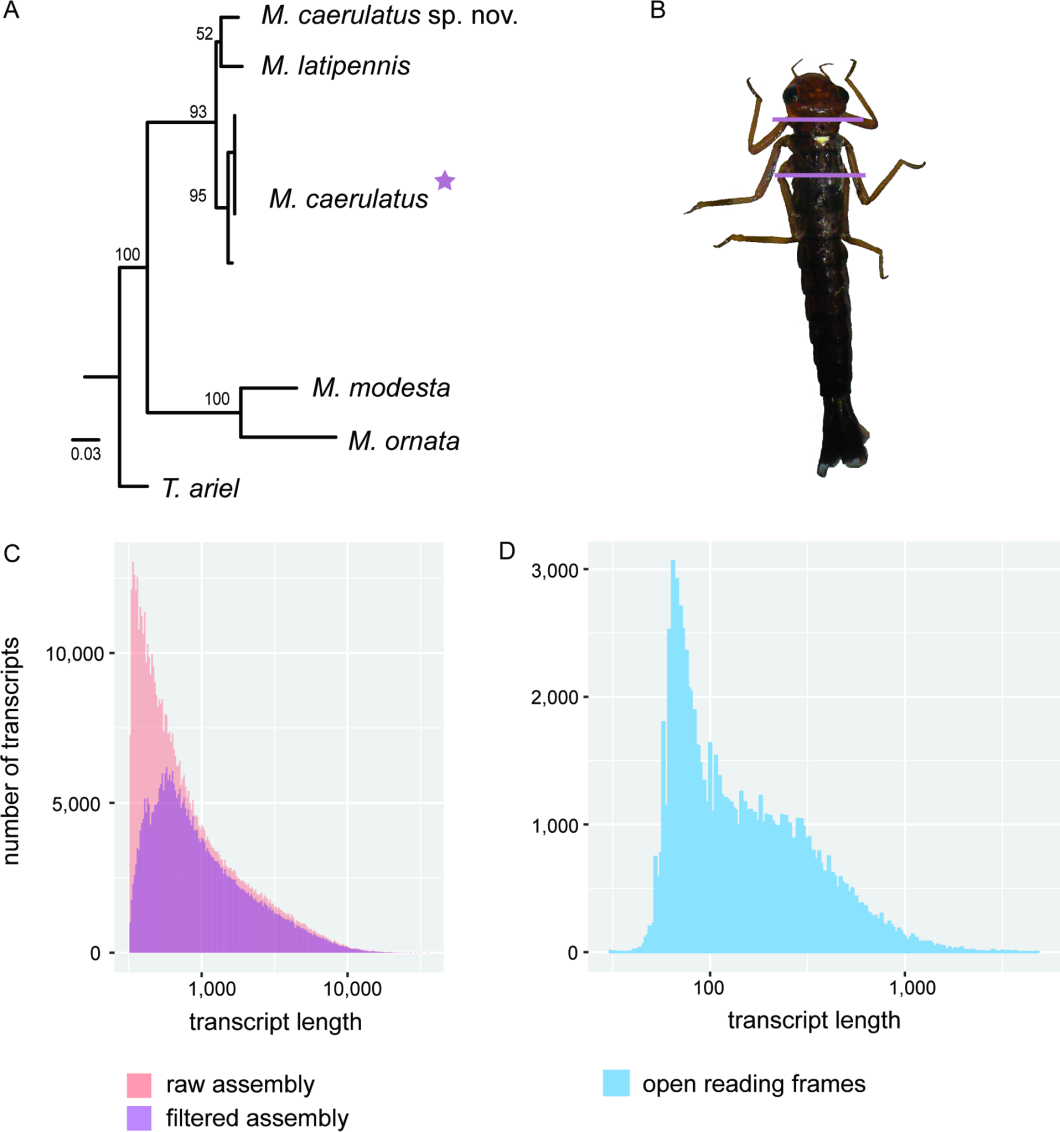
Thorax transcriptome of *Megaloprepus caerulatus*. A) Phylogeny based on the 16S rRNA gene showing the position of *Megaloprepus caerulatus* within the Pseudostigmatidae using *Teinobasis ariel* as outgroup (cf. [19]). The NCBI accession numbers are: KF895223, DQ642987, KF895193, JQ966660, KF895130, KF895162, JQ966657, DQ642983, JQ966662. B) Exemplary illustration of a *M*. *caerulatus* larva–here of about 2.5–3 cm in length. The section between the two lines indicates the tissue used for RNA extraction. C) Difference in the number of transcripts over transcript length between the raw assembly and the filtered assembly. The filtering reduced redundancy and the amount of shorter transcripts. D) Length distribution of the final predicted open reading frames. Note whereas in plot B both axes are logarithmic; in C, only the x-axis is logarithmic.

Among the oldest flying insects [14–16], Odonata (dragonflies and damselflies) with their exclusive set of bioindicator traits hold a key role as “non-model” organisms in ecological and evolutionary research [17, 18]. However, in the evolutionary-developmental context, the molecular basis of wing development and the evolution of morphological variation in odonate wings, so far received little attention. One particular species promising more insights into wing evolution is the Neotropical damselfly *Megaloprepus caerulatus* (Odonata: Zygoptera, Pseudostigmatidae), because a recent study of *Megaloprepus* revealed a radiation into at least three geographically separated cryptic species ([19], Fig 1A). These species show differences in wing shape, i.e. in wing width, the curvature of the lower wing margin and width of the blue wing band (Feindt et al. in prep). In addition, only the nominal species *M*. *caerulatus* shows sexual dimorphism in wing coloration. It has been described that modified expression patterns or signaling cascades are responsible for the variation in wing morphology, since such changes are associated with downstream responses to supposedly conserved wing-pattern genes [1, 2, 7, 20].

Integrative research on the origin of morphological variation associated with diversification in odonates is rare and hampered by a shortage of primary data. ‘*Omic*’ studies are still at their beginning and have focused so far on three species: *Enallagma hageni* [21], *Ischnura elegans* [22–25] and *Calopteryx splendens* [26]. Only one study addressed the importance of transcriptional information across embryogenesis to highlight gene sets involved in morphogenesis [24]. Undoubtedly there is a need to integrate developmental data into evolutionary research to—for example—obtain a broader knowledge of species and tissue specific expression patterns.

Thus, we here present a *de novo* transcriptome assembly from the larval thorax of *M*. *caerulatus* with the overall goal of detecting expressed genes related to wing patterning that might be relevant to the interplay between genomics, development and morphological variation. Specifically, we first focus on a high completeness of the transcriptome and catalogue the candidate wing genes in odonates found in *M*. *caerulatus*. Secondly, to portray larva-specific genes, we compared the transcriptome of *M*. *caerulatus* with that of two adult odonate species: *I*. *elegans* (Odonata: Coenagrionidae) and *Ladona fulva* (Odonata: Libellulidae).

## Material and methods

### Sample collection, RNA isolation and sequencing

One individual *M*. *caerulatus* larva (Fig 1A and 1B) was collected from a natural tree hole [27] in a lowland rain forest at the La Selva Biological Station (OTS, Organization for tropical Studies) in Costa Rica (10° 26’ N, 83° 59 W). The larva (total length = 1.96 cm) was immediately euthanized and stored in RNA*later* (Thermo Fisher Scientific Inc., USA). Prior to RNA isolation, the larva was dissected on ice to isolate the thorax (including the dorso-lateral wing buds) from the head and abdomen (Fig 1B). The tissue was frozen in liquid nitrogen and ground with a pestle. Total RNA was extracted from the thorax using TRIzol reagent (Invitrogen, USA) in combination with RNeasy Micro kit (Qiagen Inc., USA) for subsequent RNA purification. Overall quality and quantity of the isolated RNA were assessed with the BioAnalyzer 2100 (Agilent Inc., USA). Although the larva was collected into RNA*later*, some RNA degradation had occurred. Therefore, we used a TruSeq Stranded Total Library Preparation kit (Illumina, Inc., USA) for library preparation, with Ribo-Zero treatment to select preferentially for mRNA transcripts. The cDNA libraries were paired-end sequenced (2x125bp) on an Illumina HiSeq 2500 (Illumina, Inc.).

### Read cleaning and *de novo* assembly

Raw sequence reads were first checked for overall quality using a Phred-like score in FastQC [28] and, based on these results, adapters and low quality reads were removed with Trimmomatic 0.33 [29] at the Q20 level. Reads containing ribosomal RNA (rRNA) sequences were erased from the dataset to avoid mis-annotation of rRNAs as putative proteins [30] using SortMeRNA version 2.0 [31]. The Kraken taxonomic sequence classification system version 0.10.5 [32] was applied to filter out prokaryotic sequences. Those reads belong potentially to microorganisms co-inhabiting tree holes or to the microbiome of the larva. Singleton reads (where only 1 member of a read pair remained after the previous cleanup steps) were further removed before assembly.

The *de novo* assembly was conducted using Trinity version 2.0.6 [33, 34] with default parameters except for setting the strand specific flag (RF), a read normalization, and a lower limit of 300 bp on contig size. Assembly quality and completeness were evaluated in several steps. General assembly summary statistics were calculated via *TrinityStats*.*pl* [34]. As a more reliable estimator of assembly completeness we also calculated additionally the ExN50 statistic. Reads were mapped back to the assembly [35] and following Haas et al. [34] the Ex90N50 was determined. This represents the N50-value at 90% of the total normalized contigs, which is excluding contigs with a low read coverage. For an evaluation of completeness BUSCO-Benchmarking Universal Single-Copy Orthologs [36] version 1.1 was used and the RSEM-EVAL package distributed with DETONATE [37] represented our reference-free evaluation method to calculate assembly scores. Because CD-Hit [38] reduced our BUSCO scores, we finally filtered the raw assembly by applying RSEM-EVAL’s contig impact score [37]. Contigs with impact scores less or equal than zero were removed from the assembly using an in-house R script in RStudio [39] and the Bioconductor R package [40].

The cleaned raw reads are available under BioProject: PRJNA336267, BioSample: SAMN05507136 and the sequence read archive (SRA) SRR3997526. Our Trinity assembly used for all subsequent analyses is available in NCBI’s Transcriptome Shotgun Assembly database under the TSA GEXY00000000.

### Gene prediction and functional annotation

Open reading frames (ORFs) from start to stop codon on a six-frame translation were identified using TransDecoder (http://transdecoder.github.io) [34]. To further improve the ORF identification, the filtered assembly was first blasted against the arthropod data base (e-value cutoff: 1e^-5^) downloaded from UniProtKB [41]. This was followed by HMM (hidden Markov models) searches against the Pfam-A protein domain database [42] via Hmmer version 3.1 [43]. To maximize sensitivity, these results were retained as a basis for informing protein prediction in a second TransDecoder step (2-step prediction). The final predicted protein completeness was evaluated using BUSCO [36].

For functional annotation, initial sequence homology searches were performed with BLASTp (e-value cutoff: 1e^-7^) against an individually designed “insect reference data base”. This customized data base contained the arthropod protein database from UniProtKB (including SWISS-PROT and TrEMBL, [41]) and protein databases for 4 Hemiptera, 21 Hymenoptera, 3 Lepidoptera and Coleoptera, and 26 Diptera species as the closest relatives to Odonata available from NCBI (data downloaded August 3^rd^, 2015). Sequences without a hit were additionally blasted against the non-redundant database nr—RefSeq: NCBI Reference Sequence Database (downloaded June 14^th^, 2016) using BLASTp and an e-value threshold of 1e^−7^. Putative protein sequences and BLAST results were uploaded to Blast2GO [44, 45], where InterProScan [46] searches were carried out. The InterProScan and BLAST results were used for Gene Ontology (GO) term mapping (http://geneontology.org/) [47].

### Identification of key genes

The annotated transcriptome was screened for genes related to stress response, housekeeping genes, developmental genes, and genes responsible for wing development and coloration. Stress response and housekeeping genes were extracted from the annotated *M*. *caerulatus* transcriptome searching for keywords via Blast2GO [44].

To detect genes involved in insect development, reference sequences were downloaded from the Homeobox database (HomeoDB; http://homeodb.zoo.ox.ac.uk/, [48, 49]). Hereby we focused on the HOXL subclass (*Hox* genes and *Hox*-derived genes) and NKL subclass (ParaHox gene cluster), both are fractions of the largest gene class Antennapedia (ANTP class) within the homeobox genes. The HOXL subclass and NKL subclass reference sequences were blasted against the *M*. *caerulatus* transcriptome and hits were verified via local BLAST searches. In order to identify additional differences of gene expression between adults and larvae, the *Hox* gene and ParaHox gene cluster reference sequences were also blasted against the *I*. *elegans* (SRR1265958) and *L*. *fulva* (SRR1850403) transcriptomes (see section comparison with other Odonata).

Genes responsible for wing pigmentation and wing development including wing shape such as the wing gene regulatory network (wing-patterning network) and the four major wing developmental signaling pathways (Hedgehog: *Hh*, Decapentaplegic: *Dpp*, wingless: *wg* and Notch: *N*) were identified within the *M*. *caerulatus* transcriptome via reciprocal BLASTp searches. Thus reference sequences were downloaded from Swiss-Prot [41] or NCBI and blasted against the transcriptome. All potential positive hits were verified via a local BLAST search or inside B2GO [44].

### Comparison with other Odonata

The predicted open reading frames of *M*. *caerulatus* were compared to the damselfly *I*. *elegans* (SRR1265958) and to the dragonfly *L*. *fulva* (SRR1850403). Raw reads for both species were downloaded from the NCBI’s sequence read archive (http://www.ncbi.nlm.nih.gov/Traces/sra), assembled *de novo* [33, 34] and open reading frames predicted (http://transdecoder.github.io) following the steps described above, again under the strict completeness control. The Trinity assemblies for *I*. *elegans* and *L*. *fulva* are available upon request.

Overlaps were determined via comparative sequence similarity applying a reciprocal BLAST search using an in-house Perl script that reverts to BLASTp with a significant e-value of 1e^-7^. OrthoVenn [50] was further applied to categorize the transcripts into orthologous clusters. It simultaneously annotates the clusters, which were extracted to compare among the three transcriptomes.

## Results

### Sequencing and *de novo* transcriptome assembly

Sequencing generated more than 14.4 Gbp of raw data consisting of ~115 million 125 bp paired-end reads. The cleanup steps used to filter the raw reads reduced their number by ~2%, for a final set of 112 million high-quality reads (see Table 1 for a detailed trimming report).

**Table 1:** Trimming statistics using three different filtering steps.

Number PE raw reads	114,824,092.00
Read length in bp	125.00
**Trimmomatic**	
Number low quality reads	39,096.00
Percent low quality reads	0.03
Reads remaining after Trimmomatic	114,784,996.00
**SortMeRNA**	
Number rRNA reads	1,244,902.00
Percent rRNA reads	1.10
Reads remaining after rRNA removal	113,540,094.00
**Kraken taxonomic sequence classification system**	
Reads classified as contaminants	804,313.00
Percent classified as contaminants	0.70
PE reads remaining after cleanup	112,534,902.00

Using Trinity [33, 34] raw reads were assembled *de novo* into a transcriptome containing 567,572 contigs longer than 300 bp, with an N50 value of 1,956 bp (Table 2). Using Bowtie 2 [35] read support was assessed by mapping the reads back to the assembly and found that 73% of the reads mapped back in proper pairs. The Ex90N50 statistic was 2,478 bp and therefore higher than the traditional N50 measure. To evaluate the quality of the individual contigs, we used RSEM-EVAL [37], which is displaying impact scores as an estimate of read support for each contig and its contribution to the assembly. Some 84,000 low scoring contigs were removed from the assembly, reducing the assembly size to 382,606 contigs (Fig 1C). These initial assembly evaluation steps are critical in *de novo* transcriptome studies, because false positives (the inclusion of misassembled contigs) will lead to errors in gene prediction, annotation, and further downstream analyses such as expression profiling. However, false negatives (the elimination of legitimate contigs) can reduce the completeness of the transcriptome; thus, evaluations should be repeated after each filter step (Table 2). The final assembly was ~93% complete based on BUSCO’s [36] arthropod reference database of 2,675 single-copy orthologs present in >90% of the species (Table 2), which is consistent with results from other recently published insect transcriptomes (e.g. [22]).

**Table 2:** Assembly statistics during final assembly evaluation steps.

	raw assembly	filtered assembly	predicted ORFs	
**Assembly assessment parameters**				
Transcripts > 300 bp	567,572.00	382,606.00	61,560.00	
Total contig length	674,031,026.00	539,335,401.00	66,236,823.00	
Mean contig size (bp)	1,187.57	1,409.64	1,075.97	
Number of contigs > 1000 nt	175,803.00	154,692.00	21,023.00	
N50 contig length	1,956.00	2,162.00	1,605.00	
Longest contig	35,790.00	35,790.00	24,318.00	
Percent GC	38.56	38.59	45.73	
**BUSCO—annotation completeness via universal single-copy orthologous genes**				
Complete Single-Copy BUSCOs	2,236.00	2,244.00	2,173.00	
Complete Duplicated BUSCOs	1,497.00	1,406.00	1,175.00	
Fragmented BUSCOs	266.00	270.00	304.00	
Missing BUSCOs	173.00	161.00	198.00	
Complete BUSCOs in %	83.59	83.89	81.23	
**Total BUSCOs in %**	**93.53**	**93.98**	**92.60**	
**DETONATE–RSEM-EVAL’s contig impact scores**				
Score	-8,182,390,224.62	-7,955,408,062.90		
Prior score on contig sequences	-934,405,410.56	-747,677,625.16		
Expected aligned reads	39,576,297.27	39,708,827.69		
Contigs with no read aligned	84,119.00	78.00		

### Gene prediction and functional annotation

*TransDecoder*.*LongORFs* [34] identified about 93,000 potential open reading frames (ORFs) in the final *M*. *caerulatus* assembly. The homology-based second step retaining BLAST [51] and Pfam [52] search results in *TransDecoder*.*Predict* [34] resulted in a final set of 61,560 predicted proteins longer than 100 amino acids (Fig 1D, Table 2).

**Fig 2:**
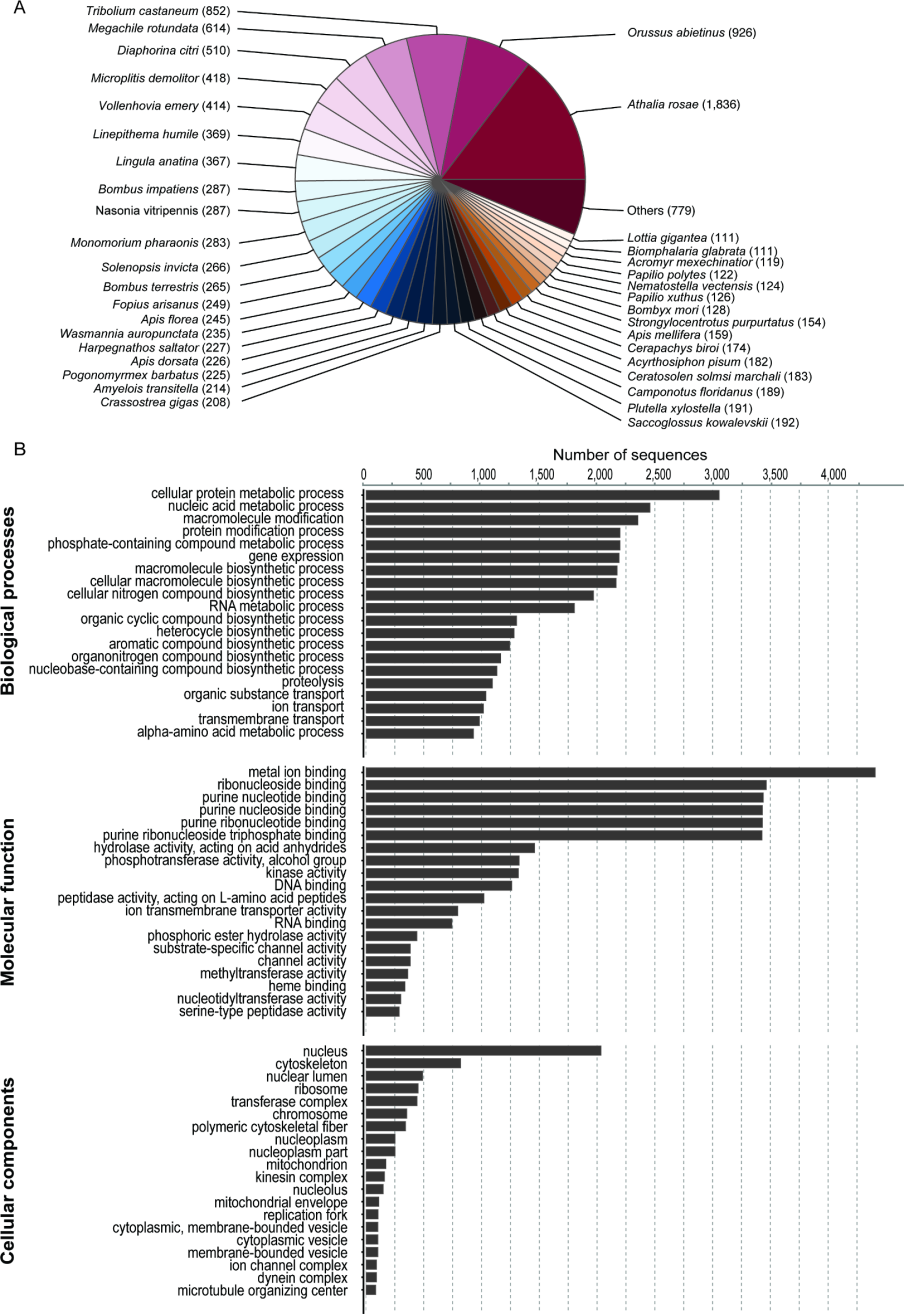
Functional annotation of the *M*. *caerulatus* transcriptome. A) Distribution of top hits shows all species to which *M*. *caerulatus* had at least 100 hits to. B) Classification of the functional annotation into the three gene ontology (GO) categories: molecular function (MF), cellular component (CC), and biological process (BP) at GO level 5. Displayed are the distribution of the top 20 GO terms and the number of sequences with the corresponding assignment.

The continuous BLASTp search against our custom ‘RefSeq’ database allowed the determination of gene functions of about 73.04% of our sequences. However, the 27% that had no hit to this database and were additionally blasted against the entire non-redundant database, which produced hits for another 1%. The top hit species distribution shows the highest number of hits against the basal Hymenopterans (Symphyta) *Athalia rosae* and *Orussus abietinus* (see Fig 2A). Accuracy of our assembly and the predicted protein coding genes were supported by consistently high e-values (42% of the blast hits had e-value >1e^-100^; see e-value distribution in S1 Fig).

Our gene ontology (GO) term assignment [47] via Blast2GO [44] revealed 78% of the putative genes had an InterPro hit, and 46% had a GO annotation (Fig 2B). Longer sequences were more likely to be annotated than shorter ones (see S2 Fig): approximately 50% of the sequences >200 amino acids were annotated, and almost all of those >500 AA.

### Identification of candidate genes

#### Stress response genes

Environmental studies on insects frequently focus on heat shock proteins (HSPs), a large and highly conserved gene family involved in protein metabolism and insect survival through their roles in protein folding and repair (e.g. [53, 54]). Under cellular stress, HSP expression levels increase and their assessment in natural environments can help identify stress adaptation under climate change or habitat fragmentation [25]. We identified 23 HSP genes and 3 general stress response genes (see S1 File for the AA sequences and gene names).

#### Housekeeping genes

Basic cell functions are controlled by housekeeping genes, expressed in every tissue under most experimental conditions (e.g. [55]) and these serve as a baseline for normalizing quantitative real-time PCR or RNA-Seq gene expression experiments. We identified the majority of housekeeping genes commonly found in insects, including the ribosomal proteins S18 and L13a as well as ATP and actin genes (S2 File).

#### Developmental genes

*Hox* genes are of particular interest as they encode transcription factors that modulate bauplan development during early embryogenesis and determiners of cell fate (e.g. [56]). With the focus on the *Antp*-class genes, we identified *M*. *caerulatus* orthologs for only three *Hox* genes, including: Antennapedia (*Antp*, Hox6-8), Ultrabithorax (*Ubx*, Hox6-8) and Sex combs reduced (*Scr*, Hox5); and one *ParaHox* gene (*Nedx*). *Hox* genes were also identified in the *I*. *elegans* and *L*. *fulva* transcriptomes. An alignment of these first full-length homeodomain amino acid sequences for our 16 detected *Hox* and *ParaHox* genes in Odonata is shown in S3 File.

#### Wing genes

Insect wing development is controlled by the wing-patterning network (wing gene regulatory network) in which the *Hox* genes *Scr* and *Ubx* act jointly with cell signaling molecules, selector genes and transcription factors to modulate wing morphogenesis, differentiation and growth [2, 57]. These signaling molecules are further grouped into four main signaling pathways: Hedgehog (*Hh*), Decapentaplegic (*Dpp*), wingless (*wg*) and Notch (*N*) constituting overarching structures [5, 58, 59]. In addition, the wing-patterning network influences wing coloration, as developmental gene expression determines the activity of subsequent pigment genes (e.g. [2]).

We were able to identify most representatives of the pigmentation genes, 14 of the 21 genes described so far for the wing-patterning network, and the four main developmental signaling pathways. For the latter, we could discover 6 genes related to accurate cell differentiation and growth in the Hedgehog pathway, 8 genes that are included in the cell fate determination by the Notch pathway, 9 genes associated with the development of wings in the wingless pathway and 3 genes could be connected to dorsal/ventral patterning and development of the wing epithelia in the Decapentaplegic pathway (see Table 3 for a complete overview of wing genes and their related pathways and S4 File for the corresponding amino acid sequences). The reciprocal BLASTp searches against well-annotated patterning genes revealed an additional 19 genes described in wing coloration and general pigmentation studies [2, 4, 60–64]. We detected pigmentation genes from the melanin pathway (*yellow*, *black*, *tan*, *pale*), the pteridine pathway (*henna*, *rosy*, *prat*), the ommochrome pathway (*vermillion*, *white*, *scarlet*) and pigment granule genes (*dor*, *garnet*). We also found phenol oxidases (*PO*), which contribute to melanization among other functions [6, 64] and the Ecdysone receptor (*EcR*), a hormone involved in wing growth [65, 66].

**Table 3:** Selection of genes responsible for wing development in insects. Genes of the four major signaling pathways and functionally related genes (“Others”) are arranged in the according columns. Members of the wing-patterning network [2, 57] known to be associated with a specific signaling pathway are shown in the grey row [5, 9, 58, 59, 67–72]. Genes identified in the *M*. *caerulatus* transcriptome are shown by bold; while the corresponding amino acid sequences and unigene IDs are given in S4 File and a description of primary gene functions are in the S1 Table. Genes that could not be identified in the *M*. *caerulatus* transcriptome are shown in black font.

	Hedgehog	Decapentaplegic	Notch	Wingless	Others	*Homeobox* genes
**Wing-patter-ning net-work**	**Hedgehog (*Hh*)**	**Decapentaplegic (*Dpp*)** **Optomotor-blind (*bi [omb]*)** **Spalt major (*salm*)** Spalt related (*salr*)	**Serrate (*Ser*)** **Cut (*cut*)** **Achaete (*ac*) / Scute (*sc*)**	**Wingless (*wg*)** **Scalloped (*sd*)** Vestigial (*vg*)	**Blistered (bs) syn: Serum Response Factor (*srf*)** **Spitz (*spi*)** Apterous (*ap*) Engrailed (*en*) Escargot (*esg*) Snail (*sna*)	**Ultabithorax (*Ubx*)** **Sex combs reduced (*Scr*)** **Extradenticle (*exd*)** Abdominal A (*Abd-A*) Distal-less (*Dll*)
	**Patched (*Ptc*)** **Smoothened (*Smo*)** **Cubitus interruptus (*ci*)** **Costa (*cos*)** **Fused (*Fu*)** **Suppressor of fused (*sufu*)**	**Mothers against Dpp (*mad*)** **Brinker (*brk*)** Medea (*Med*) Glass bottom boat (*Gbb*) Saxophone (*sax*) Punt (*put*) Thickveins (*tkv*)	**Notch (*N*)** **Delta (*Dl*)** **Hairless (*H*)** **Suppressor of hairless (*Su(H)*)** **Mastermind (*mam*)** **Notchless (*Nle*)** **Fringe (*fng*)** **Hairy (*h*)** Nipped-A (*Nipped-A*)	**Frizzled** (*fz*, ***fz2***, *fz3*, *fz7*) **Disheveled (*dsh*)** **Armadillo (*arm*)** **Pangolin (*pan*)** **Wingful/Notum (*Wf*)** **Naked cuticle (*Nkd*)** **Nemo (*Nmo*)** **Axin (*Axn*)** **Adenomatous polyposis coli (*Apc*)** Arrow (*arr*) Dally (*Dlp*) Shaggy (sgg) GDI interacting protein 3 (*Gint3*)	**Homothorax (*hth*)** **Epidermal growth factor receptor (*Egfr*)** **Rhomboid (*rho*)** **Four-jointed (*fj*)** **Deadpan (*dpn*)** **Rap1 GTPase (*Rap1*)** **Small wing (*sl*)** **UV-resistance associated gene (*Uvrag*)** **Capricious (*caps*)** **Rickets (*rk*)** **Tolkin (*tok*)** Teashirt (*tsh*) Nubbin (*nub*) Pannier (*pnr*) Fat (*ft*) Gilgamesh (*gish*) Homeodomain interacting protein kinase (*Hipk*) Knirps (*kni*) Extra macrochaetae (*emc*) Net (*net*) Ventral veins lacking (*vvl*)	

### Comparison with other Odonata

Although direct comparative transcriptome analyses struggle with differences in sample preparation (e.g. different tissue collection, developmental stages, etc.) and by the difficulty of accurate ortholog detection, first comparisons amongst well-annotated sequences are appropriate for a selected set of questions (e.g. [13, 73]). Here, we compared our findings with the transcriptomes of *I*. *elegans* and *L*. *fulva* to search for unique gene expression. The damselfly *I*. *elegans* belongs to the Coenagrionidae—a sister family to the Pseudostigmatidae to which *M*. *caerulatus* is associated—while the more distantly related dragonfly *L*. *fulva* belongs to the suborder Anisoptera.

**Fig 3:**
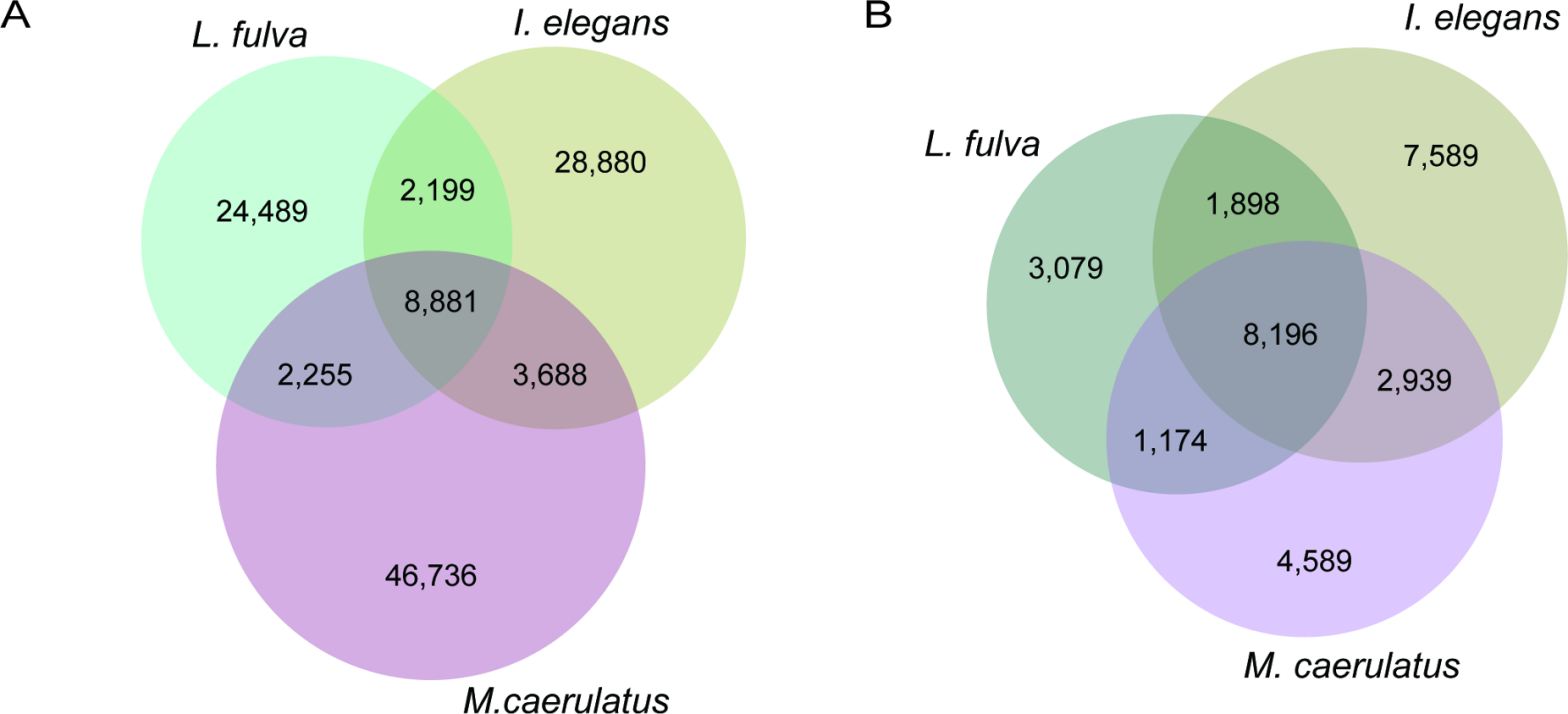
Comparisons among three odonate transcriptomes based on the open reading frames: *Ladona fulva*, *Ischnura elegans*, and *M*. *caerulatus*. A) The overall sequence similarity identified via reciprocal blast search among transcriptomes presented in a Venn diagram shows a greater number of overlapping genes between *M*. *caerulatus* and *I*. *elegans* than between the dragonfly *L*. *fulva* and the two damselflies. B) Overlapping orthologous gene clusters (OrthoVenn). Both analyses show a similar sized overlap among species.

Our results reflect these relationships in that the highest overall sequence similarity is represented by the 12,569 reciprocal best hits between *M*. *caerulatus* and *I*. *elegans*. In the *M*. *caerulatus* / *L*. *fulva* search, 11,136 reciprocal best hits were obtained, similar to the results for *I*. *elegans* / *L*. *fulva* (Fig 3A). The comparison of both overlapping and unique orthologous clusters for each species and species pair showed a similar result (Fig 3B). Using OrthoVenn [50] we retrieved a total of 29,464 clusters, with 8,196 clusters containing genes from all three species. Of these, a functional annotation was available for 4,810 clusters.

To gain insights into larva-specific genes we focused on the unique orthologous clusters of *M*. *caerulatus*. In total 4,589 were detected, but only 1,168 clusters had a usable annotation. Those clusters were related primarily to general cell functions such as phosphorylation (serine/threonine-protein kinases, cytochrome oxidases) and signal transduction (receptor tyrosine kinases). Among potential larva-specific transcripts, we identified the following related to wings and general development: (i) *encore* regulates dorso-ventral polarity in embryos and larvae; (ii) *flightless-1* plays a structural role in indirect flight muscle; and (iii) *krueppel* is involved in gap class segmentation. Other interesting findings were the *O-mannosyl-transferase 2* that is responsible for somatic muscle development and the *Ryanodine receptor 44F* which is involved in proper muscle function, i.e., in larval body wall muscles, and is therefore essential for larval development [74, 75].

## Discussion

At the base of flying insects, Odonata have a long-standing record in ecological and evolutionary research. This head start should encourage their future role as non-model systems in integrative genomic research. The first transcriptome profiling of a larval tissue from *Megaloprepus caerulatus* represents a step towards this direction to study wing development and evolution, and speciation.

### Transcriptome assembly and functional annotation

Odonate genomes are among the larger known genomes within winged insects [76], and dragonfly and damselfly transcriptomes likewise appear to be larger than those of prominent model species such as Diptera or Lepidoptera. Our assembly of raw sequences resulted in over 500,000 putative transcripts, which were reduced via strict evaluation to 61,560 high quality protein-coding genes. This number and the ExN50 expression value reflect some redundancy, but the transcriptome size is comparable to that of other odonates (e.g. [21, 22]). Furthermore, in comparison to previous odonate studies, function could be assigned to a greater number of genes which may reflect growing resources in genomics and the use of customized and frequently updated reference databases [77].

Beyond this, increasing tissue diversity should in turn increase the number of genes sequenced and annotated, but difficulties with the assembly of heterozygous sequences can limit the quality of the reconstructed transcripts and thereby impair the reliability of BLAST results. In spite of the comparatively successful assignment of function, some 25% of the putative proteins lack annotation. Some of these genes are probably misassembled transcripts that do not actually exist or, alternatively, represent odonate- or *Megaloprepus*-specific proteins that simply lack homologous sequences in current databases.

### Candidate genes

Little is known about developmental genes such as *Hox* genes or those responsible for the pathways of wing development and coloration in odonates (but see [56]). In accordance with our expectations we found three *Hox* genes in the larval thorax. Interestingly, six *Hox* representatives could be detected in the adult *I*. *elegans* (S3 File), suggesting that *Hox* expression may be of functional importance in adults as well as larvae. Two of the *Hox* genes identified in *M*. *caerulatus* are involved in important wing traits: *Scr* suppresses wing development in the prothorax [1], while *Ubx* controls hind wing identity [72] and is an important modulator in the wing-patterning gene regulatory network [2]. It acts as a selector gene, influencing morphological characters such as wing venation and regulates wingless (*wg*), splat (*sal*) and vestigial (*vg*) in opposing mechanisms [71]. In *Drosophila* it facilitates the development of halteres and in *Tribolium* the sclerotized fore wings [72].

The development of wings and their shape is controlled by the wing-patterning network through the modulation of gene expression [6, 78]. It was originally described in *Drosphila melanogaster* and is supposedly largely conserved across holometabolous insects [2, 57]. However, in hemimetabolous insect orders information on wing differentiation across larval stages is limited [79]. In the *Megaloprepus* transcriptome, we identified 14 genes from the wing-patterning network. So far *Dpp* has been described to inhibit *Dll* (Distal-less) in an early stage of the signaling cascade within the wing-patterning network, but later in the development of imaginal wing discs it activates *omb* (Optomotor-blind), *sal* (spalt) and *vg* (vestigial) to shape cell growth, vein positioning and intervein cell differentiation [2, 57]. In the pupal stage of holometabolous insects, a significant reorganization of tissues and organs takes place, while hemimetabolous insects undergo a more gradual developmental transition. Thus some of the mechanisms of wing development in Odonata most likely differ from those of holometabolous insects, and further investigation of the timing and related genes may shed light onto the developmental changes that characterize the bauplan transition to holometaboly [1].

Wing coloration in odonates is highly variable across species. Some have only a colored pterostigma or different sized wing spots, while some other species show entirely colored wings. We identified 19 genes related to insect pigmentation. Furthermore, our data showed a higher relative expression of both, the *phenol oxidases* and *yellow* in comparison to the house keeping genes (S5 File). This could be an indication of polymerization of cuticular pigments following larval molt. However, since the larva was collected from its natural environment, this remains an assumption pending controlled experiments under laboratory settings to identify the genes and pathways responsible for coloration. Targeted RNA sequencing in parallel with in situ hybridization studies would thus provide deeper insights into gene expression during the course of odonate development.

Finally, some of the wing and developmental genes that were not identified in our analysis may simply lack expression at the time of collection, but most likely may indicate modified signaling pathways. However, we suggest that the genes identified here reflect an early stage of wing development, also because the *Megaloprepus* larva used bore visible wing buds on its thorax. In our comparison between the larvae and the two adult odonates, we found aside of the wing genes, proteins known as essential for larval development (*O-mannosyltransferase*, *Ryanodine receptor 44F*). However, many of those ~4,500 transcripts found only in *M*. *caerulatus* may be species-specific rather than larva-specific.

## Conclusion

*Megaloprepus caerulatus* has a longstanding record in ecological and evolutionary research [19, 80–84]. The *de novo* transcriptome presented here is the first genomic resource for Neotropical odonates and may hopefully enhance future genomic research in odonates. For *M*. *caerulatus* comparative studies at different developmental stages involving the newly discovered species might reveal mechanisms of wing shape divergence, demographic patterns, micro-evolutionary changes and genomic regions under selection in changing environments.

Because of its close ecological association with Neotropical old growth rainforests, high vulnerability to climate shifts and forest disturbances, *Megaloprepus* is an effective bioindicator of the history (and future) of old growth rainforests. Genomic monitoring of key genes combined with ecological data could provide early insights into the effects of environmental changes.

## Supporting information

S1 Table Genes related to wing development and coloration in insects. Included are the descriptions of the main functions for genes involved in pigmentation (S1A) and genes of the wing-patterning gene network (GRN; S1A) found in *M*. *caerulatus* including their Unigene IDs and FPKMs.
(PDF)

S1 Fig E-value distribution for BLAST hits against *Megaloprepus'* predicted proteins. (TIF)

S2 Fig Length distribution of annotated sequences within the *M*. *caerulatus* transcriptome. (TIF)

S1 File Amino acid sequences of *M*. *caerulatus* stress response genes. (FASTA)

S2 File Amino acid sequences of *M*. *caerulatus* house keeping genes. (FASTA)

S3 File Homeobox genes (HOXL subclass and NKL subclass) homeodomain amino acid alignment for *I*. *elegans*, *L*. *fulva* and *M*. *caerulatus*. (FASTA)

S4 File Amino acid sequences of wing genes found in *M*. *caerulatus*. Included are representatives of the wing-patterning gene network and the four signaling pathways: Notch (N), wingless (wg), Decapentaplegic (Dpp) and Hedgehog (Hh), and pigmentation genes.
(FASTA)

S5 File In silico quantification of *Megaloprepus* expression levels. (PDF)
