# Supplementary material for: Transcriptome profiling with focus on potential key genes for wing development and evolution in Megaloprepus caerulatus, the damselfly species with the world's largest wings
Source: PLoS One. 2018 Jan 12;13(1):e0189898. doi: 10.1371/journal.pone.0189898 (PMC5766104; doi:10.1371/journal.pone.0189898)
Supplement: S1 Table — Included are the descriptions of the main functions for genes involved in pigmentation (S1A) and genes of the wing-patterning gene network (GRN; S1A) found in M. caerulatus including their Unigene IDs and FPKMs. (PDF) [file pone.0189898.s001.pdf]

S1A Table. Summary of genes involved in pigmentation of insect wings and other body parts identified within the *M. caerulatus* transcriptome. For each gene the corresponding Unigene ID, FPKM values and their one of their main functions are shown.

gene	Unigene ID	read count	FPKM	Main function
Aristaless (*al*)	TR167515	8	0.77	*Homeobox* protein, precedes *wg* expression - wing pattern evolution
Black (*b*)	TR93729	12	0.65	Cysteine sulfinic acid decarboxylase, melanin pathway
Ebony (*e*)	TR1248	35	1.40	Inhibits melanization in non-black wing regions
Tan (*t*)	TR192472	44	1.81	Melanin pathway
Yellow (*yellow*)	TR182547	2,205	61.44	Melanin pathway
	TR183295	2,866	71.30	
Ecdysone Receptor (*EcR*)	TR215139	1,262	41.31	Ligand-dependent transcription factor, steroid receptor super family
Araucan (*ara*)	TR162214	32	2.24	Transcription factor, imaginal disc-derived and wing vein specification
Henna (*Hn*)	TR231264	874	24.74	Pteridine pathway - regulates phenylalanine
bric a brac 1 (*bab*)	TR165906	61	3.00	Axis patterning, involved in female specific pigmentation in *Drosophila* sp.
bric a brac 2 (*bab*)	TR206843	149	8.67	
Dor (*dor*)	TR165868	1,574	28.41	Pigment granule gene, vacuolar protein sorting-associated protein 18 homolog
Pale protein (*ple*)	TR106565	217	4.18	Tyrosine 3-monooxygenase, melanin pathway
Phenoloxidase 2 (*PO*)	TR158701	32,165	606.37	Melanization, final polymerization cuticular pigments, immune response
	TR200723	10,582	181.28	
	TR201540	441	8.99	
Rosy (*ry*)	TR177287	1,035	13.73	Xanthine dehydrogenase, pteridine pathway
	TR202403	136	3.21	
White (*w*)	TR203851	93	4.56	Ommochrome pathway - ommochrome precursor transporter gene
Scarlet (*st*)	TR217941	774	23.58	Ommochrome pathway, transport of pigment precursors
	TR182410	189	4.21	
Garnet (*g*)	TR16501	89	4.20	Pigment granule gene, organ pigmentation
Mal (*mal*)	TR151410	97	2.23	Molybdenum cofactor sulfurase, cofactor for synthetic processes: essential for xanthine dehydrogenase (XDH) and aldehyde oxidase (ADO)
	TR151410	35	1.51	
Prat (*Prat*)	TR15147	21	1.54	Amidophosphoribosyltransferase, purine metabolism, pteridine pathway
Vermillion (*v*)	TR155117	845	30.07	Ommochrome pathway, ommochrome enzyme gene, brown eye pigment, tryptophan 2,3-dioxygenase

S1B Table. Genes of the wing-patterning gene network found in *M. caerulatus* with their corresponding Unigene ID, FPKM values and some of their main functions.

gene	Unigene ID	read count	FPKM	Main function
Hedgehog (*Hh*)	TR219975	265	10.63	Short range signaling molecule, embryonic segmentation, segment polarity
Decapentaplegic (*Dpp*)	TR23365	19	1.98	Long range signaling molecule; embryonic dorsal/ventral patterning; boundaries between appendage compartments
	TR199155	67	2.56	
Optomotor-blind (*bi* [*Omb*])	TR174327	226	20.03	Transcription factor, wing differentiation and wing formation
Spalt major (*salm*)	TR119349	79	2.93	Transcription factor, *Dpp* signaling, controls vein-specific expression of knirps and iroquois gene complex - controls position of the wing veins
	TR211792	132	6.63	
Serrate (*Ser*)	TR178981	1,344	28.57	Short range signaling molecule, notch ligand; initiation of wing margin (dorsal-ventral) by controlling cell proliferation
Cut (*cut*)	TR148092	98	3.88	Selector gene (homeodomain transcription factor), wing and follicle cell morphogenesis
Achaete / Scute (*ac/sc*)	TR159427	610	20.65	Transcription factor, involved in the determination of the neural fate; (scute alone responsible for sex determination)
Wingless (*wg*)	TR153309	159	9.53	Long range signaling molecule, segment polarity gene, wing morphogenesis, mediating dorso-ventral compartment boundary
Scalloped (*sd*)	TR191046	573	19.87	Selector gene (homeodomain transcription factor), downstream effector molecule (wingless pathway)
Blistered [Serum Response Factor] (*bs* [*srf*])	TR175967	390	15.56	Selector gene (homeodomain transcription factor), vein / intervein formation
	TR175378	67	2.22	
Spitz (*spi*)	TR160328	60	2.67	Ligand for the epidermal growth factor receptor, differentiation of ommatidial cell types
Ultabithorax (*Ubx*)	TR214266	21	1.88	Selector gene (*Hox* gene), 'correct' dorsal and ventral appendage patterning in the third thoracic segment
Sex combs reduced (*Scr*)	TR157439	65	4.37	Selector gene (*Hox* gene), first thoracic segment development
Extradenticle (*exd*)	TR187890	498	31.83	Transcription factor, interacts with homeotic proteins for specificity of homeotic protein binding to DNA - cofactor of *Ubx*, transformation of segmental identities
