# Supplementary figures and images for: Transcriptome profiling with focus on potential key genes for wing development and evolution in Megaloprepus caerulatus, the damselfly species with the world's largest wings
Source: PLoS One. 2018 Jan 12;13(1):e0189898. doi: 10.1371/journal.pone.0189898 (PMC5766104; doi:10.1371/journal.pone.0189898)

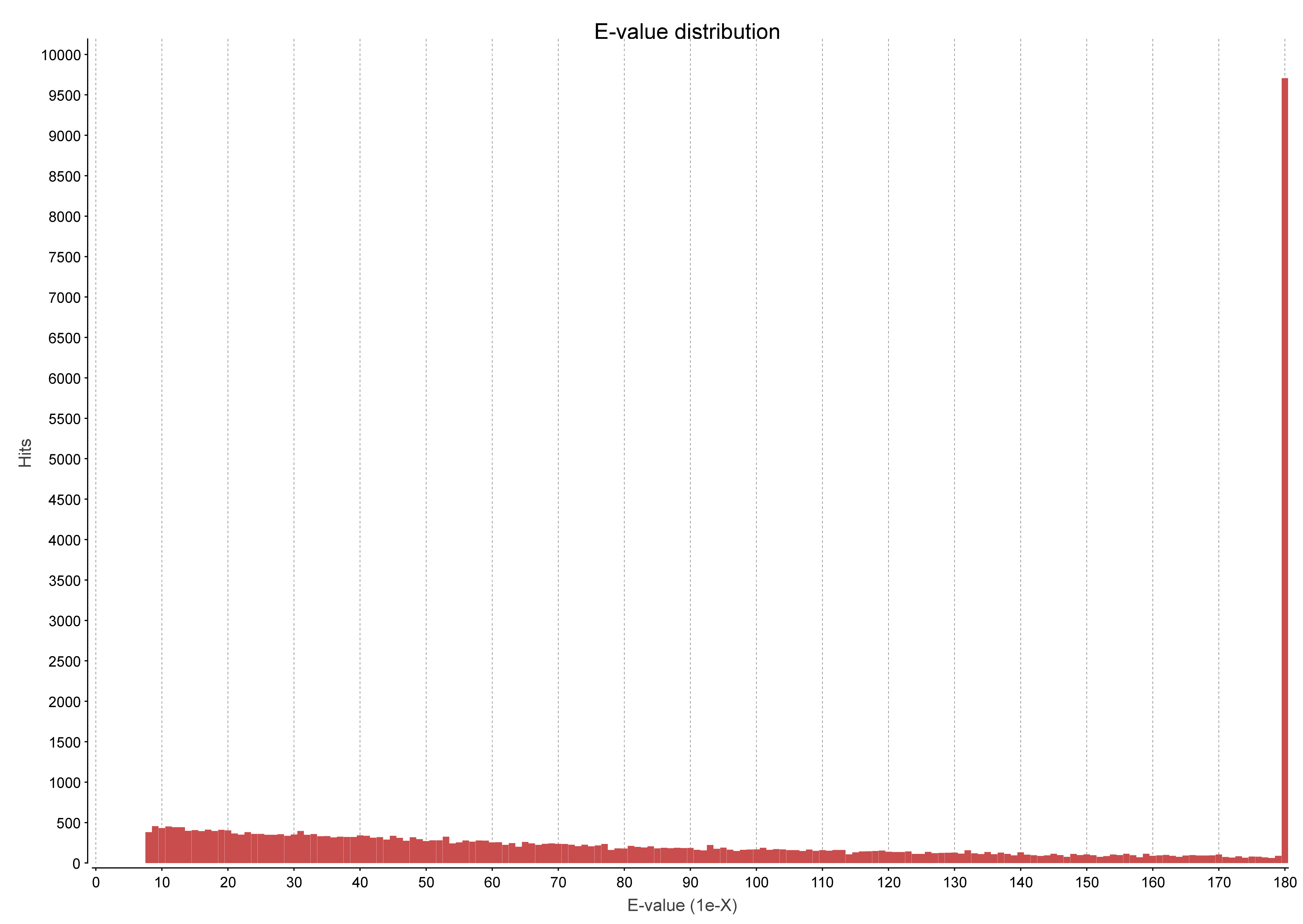

Supplement: S1 Fig — (TIF) [file pone.0189898.s002.tif]

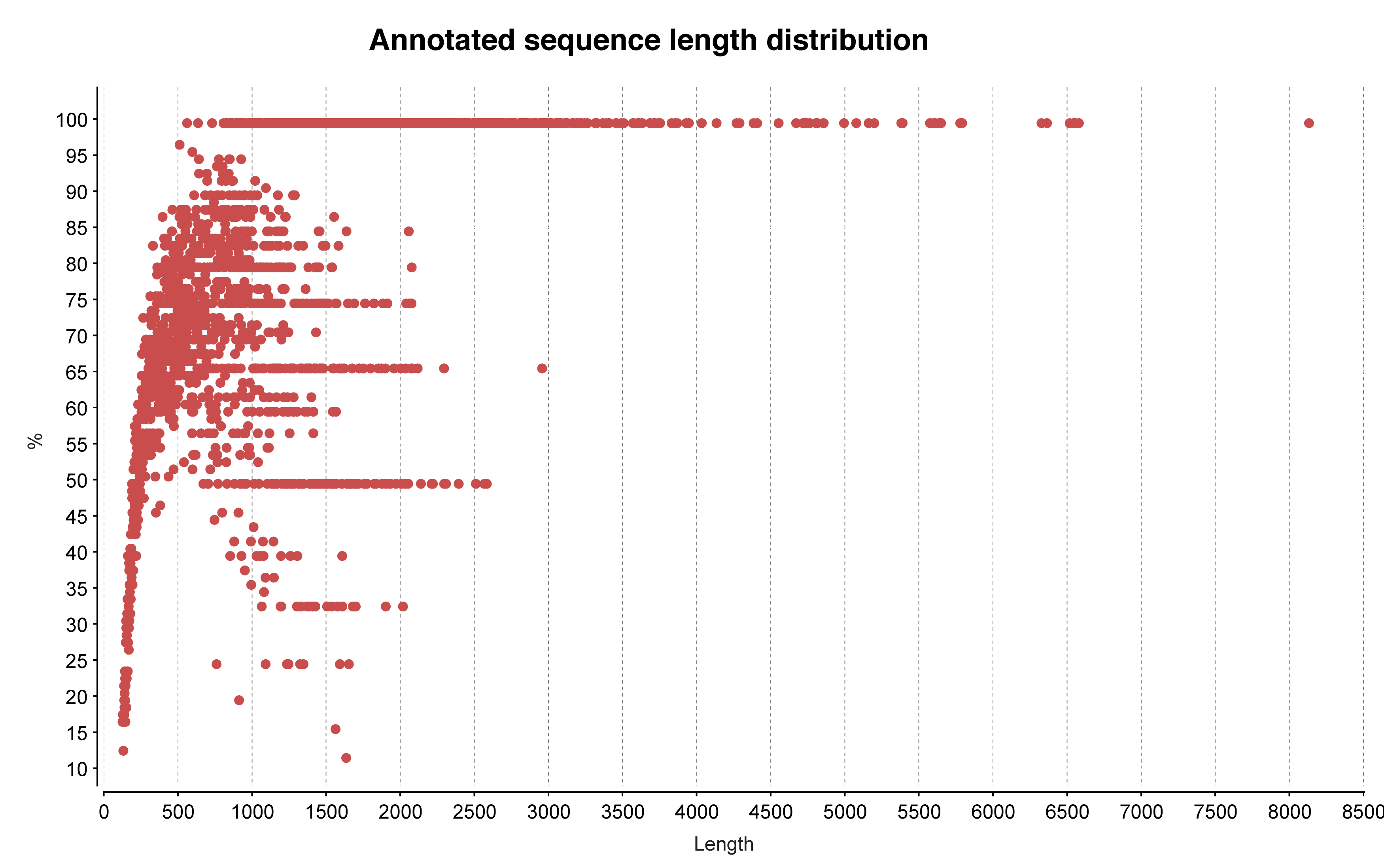

Supplement: S2 Fig — (TIF) [file pone.0189898.s003.tif]
