# Supplementary material for: Transcriptome profiling with focus on potential key genes for wing development and evolution in Megaloprepus caerulatus, the damselfly species with the world's largest wings
Source: PLoS One. 2018 Jan 12;13(1):e0189898. doi: 10.1371/journal.pone.0189898 (PMC5766104; doi:10.1371/journal.pone.0189898)
Supplement: S5 File — (PDF) [file pone.0189898.s008.pdf]

S5 File. In silico quantification of *Megaloprepus* expression levels

Although we did not design this as an expression study, transcripts with very high or very low read coverage can give a gross indication of comparative expression levels. In order to detect these, we calculated FPKM (fragments per kilobase of transcript per million mapped reads) for the functionally annotated proteins following the suggestions of [1]. Expression levels ranged from 1 to over 1 million, suggesting a wide range of expression involving few highly expressed genes. The 50 transcripts with the highest read counts were mostly giant muscle proteins, such as titin isoforms, and twitchin, which belong to the titin/connectin superfamily. A deeper look to the wing genes is depicted in S5B. Future expression studies with appropriate biological replication are needed to potentially connect these patterns to developmental processes.

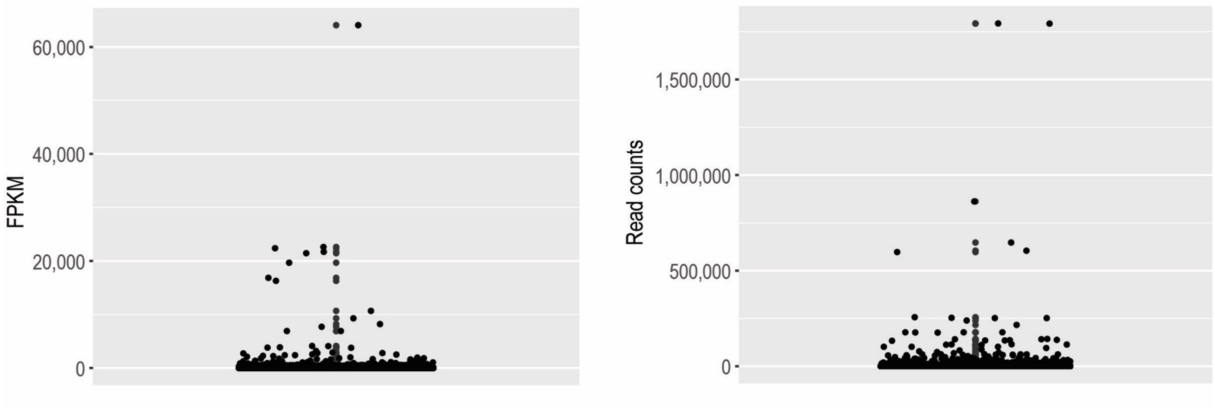

S5B. FPKM (fragments per kilobase of transcript per million mapped reads) and read count of annotated transcripts of the *M. caerulatus* transcriptome.

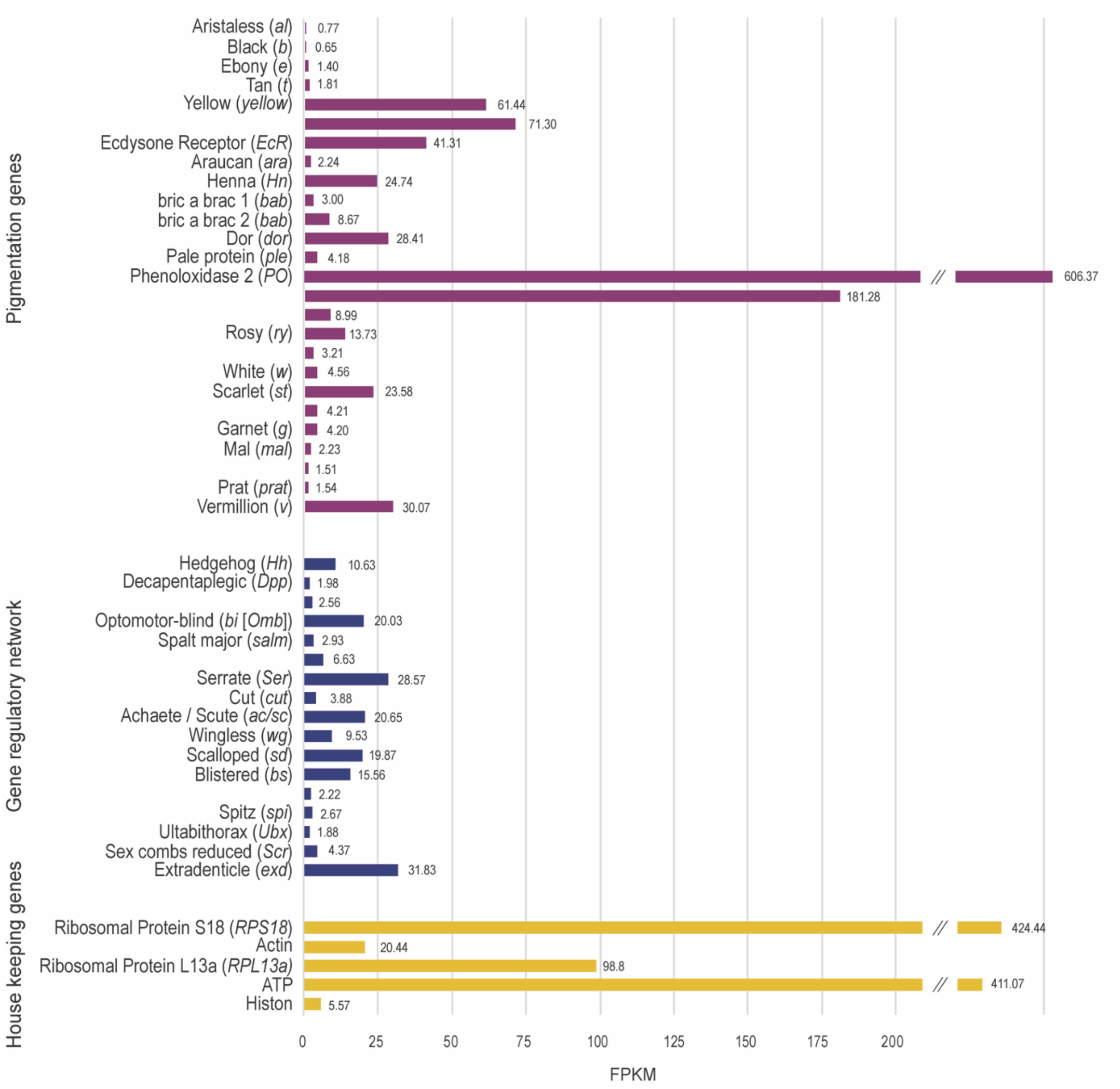

S5B. Genes related to wing pigmentation (A) and the gene regulatory network (B) identified within the *M. caerulatus* transcriptome. Shown are relative expression values per gene (fragments per kilobase of transcript per million mapped reads: FPKM) relative to a selection of house keeping genes.

Reference

1. Mehr SFP, DeSalle R, Kao H-T, Narechania A, Han Z, Tchernov D, et al. Transcriptome deep-sequencing and clustering of expressed isoforms from *Favia* corals. BMC genomics. 2013;14(1):546.
